# Predictors of hearing recovery in patients with severe sudden sensorineural hearing loss

**DOI:** 10.1186/s40463-017-0207-1

**Published:** 2017-04-04

**Authors:** Daniel Weiss, Armin Julius Böcker, Mario Koopmann, Eleftherios Savvas, Matthias Borowski, Claudia Rudack

**Affiliations:** 1 Department of Otorhinolaryngology, Head and Neck Surgery, University of Münster, Kardinal-von-Galen-Ring 10, Münster, 48149 Germany; 2 Practice for Otorhinolaryngology, Head and Neck Surgery, Meckenemstrasse 26, Bocholt, 46395 Germany; 3 Institute of Biostatistics and Clinical Research, University of Münster, Schmeddingstrasse 56, Münster, 48149 Germany

**Keywords:** Sensorineural hearing loss, Prognosis, Risk factors

## Abstract

**Background:**

Sudden sensorineural hearing loss (SSHL) is a disease, which severely affects the patient’s social and relational life. The underlying pathomechanisms have not been finally clarified yet and outcome is not predictable.

**Methods:**

We conducted a retrospective study in order to identify parameters that influence hearing recovery. The data base contains results of basic otoneurological tests and clinical parameters of 198 patients with idiopathic SSHL of at least 60 dB in at least four frequencies, diagnosed and treated at the University Hospital of Münster, Germany, between 1999 and 2015. Hearing recovery was measured by pure tone audiometry.

**Results:**

Multivariate linear and logistic regression analyses indicate that the chance as well as the magnitude of hearing recovery is higher for patients with normal caloric testing than for patients with pathological caloric testing. However, for the subgroup of patients who attained a hearing recovery, the caloric testing result was not found to influence the magnitude. Instead, the magnitude was noticeably lower for patients within this subgroup who had a previous hearing loss. Furthermore, we found indications that the magnitude is higher for men than for women and that receiving a high-dose steroid therapy is associated with a higher chance and magnitude of a hearing recovery.

**Conclusions:**

We conclude that SSHL associated with disorders of the vestibular system or previous hearing loss represent special sub-entities of SSHL that may be caused by unique pathophysiological mechanisms and are associated with worse outcome. Furthermore, our data support the importance of elevated dosage of steroids in SSHL therapy.

## Background

Sudden, sensorineural hearing loss (SSHL) is an acute, mostly unilateral dysfunction of the inner ear that is characterized by sudden onset and potential progression to complete deafness. Elderly people are primarily affected, but, it can occur at every age. While the estimated incidence is 10-20/100.000/year, the true incidence is probably much higher since it increases in industrialized countries and many cases are often misdiagnosed or regarded as age-related unavoidable fate [1–3]. Hearing impairment can be accompanied by tinnitus, ear fullness and/or vertigo [4]. The etiology of SSHL is mostly unknown and is therefore often referred to as idiopathic. Less than one third of all cases can be definitely attributed to haemorheological disturbances or viral infections, and less common to autoimmunological mechanisms [1, 4–8]. While some experimental and clinical trials could link classical cardiovascular risk factors to the development and outcome of SSHL, others could not [9–14]. Clinically, according to pure-tone audiometry, SSHL can either affect only the low, the high, or the medium frequencies, or it can affect all frequencies (pancochlear) with varying degrees up to deafness. Each of these forms may represent a distinct underlying entity [5, 15]. In individual cases of pancochlear hearing loss a perilymph fistula (PLF) may play an etiological role. A PLF can be caused by external or internal factors (e.g., head injury, scuba diving, lifting) which lead to a relative change in ambient, middle ear, or intracranial pressure, or it can occur spontaneously [16–18]. Patients with PLF mostly present with hearing loss, vertigo, tinnitus and subjective and/or objective fistula symptoms (e.g. subjective disequilibrium/nystagmus when positive or negative pressure is applied to the external ear).

In general, no effective treatment option is available for the majority of SSHL patients and many patients do not completely recover. The most important innovations in SSHL therapy in the last few decades are apheresis of fibrinogen and low density lipoproteins (LDL) and administration of steroids via intratympanic routes [19, 20]. New treatment options are urgently needed since the effectiveness of steroids (oral or systemic) in the treatment of SSHL remains unproven [21–23].

The outcome of SSHL, especially the idiopathic form, is not predictable. In the last few decades, numerous studies have attempted to establish a relationship between certain accompanying symptoms or findings, e.g. in the otoneurological or general examination, and the degree of hearing improvement with, however, often different results [24–48].

Therefore, the present retrospective study was initiated to further explore the impact of basic clinical parameter on chance and magnitude of hearing recovery after SSHL in order to detect potential variables that predict outcome.

## Methods

### Patients and otoneurological testing

In a retrospective approach we identified patients with an acute, unilateral SSHL ≥60 decibel (dB) in at least 4 frequencies between 125 and 8000Hz compared to the healthy ear. All patients were diagnosed and treated at the Department of Otorhinolaryngology, Head and Neck Surgery at the University Hospital of Münster, Germany between 1999 and 2015. By entering appropriate ICD-10 codes into a digital hospital information system (Orbis, AGFA), all eligible patients could be identified. Next, each individual patient record was reviewed for the requirements of this study. If they fulfilled the inclusion criteria, further evaluation was started. In all cases hearing loss was measured by pure-tone audiometry. Pure tone air and bone conduction threshold audiometry (with and without masking of both ears) was performed by certified and experienced audiometrists during the patients’ first visit of the outpatient care unit. Accompanying tinnitus was characterized audiometrically by determination of tinnitus loudness and frequency. Hearing recovery was calculated by comparing the pure-tone thresholds of the initial visit and the last investigation (on average 8–12 weeks after the initial visit). Hearing improvement was registered in dB in six frequencies: 250Hz, 500Hz, 1000Hz, 2000Hz, 3000Hz and 4000Hz. Possible underlying pathologies were tried to identify by otorhinolaryngological examination including ear microscopy, magnetic resonance imaging and serological blood investigations for herpes simplex virus (HSV), varizella zoster virus (VZV) and borrelia burgdorferi infections. All patients were asked for possible anamnesis of familial deafness, chronic otological history, trauma, previous ear surgery, or prior sudden deafness. Vestibular examination recorded spontaneous nystagmus, gaze nystagmus, positional nystagmus, caloric vestibular test and perilymph fistula test. Patients’ medical chart was evaluated for basic clinical parameters.

### Dataset

The dataset contains the results of basic otoneurological tests and several clinical parameters of 198 patients. The patient’s overall magnitude of hearing improvement (in dB) is expressed as the weighted mean of the hearing improvement in the six frequencies 250Hz, 500Hz, 1000Hz, 2000Hz, 3000Hz and 4000Hz, where the weights are 0.1, 0.2, 0.2, 0.2, 0.2 and 0.1. (Hence, the weight of the two extreme frequencies is half the weight of the four medium frequencies.) According to Siegel’s criteria, a hearing recovery is defined as an overall magnitude of hearing improvement of at least 15 dB [49].

Furthermore, the dataset includes several clinical parameters that are investigated w.r.t an influence on the chance and the magnitude of a hearing recovery: age at diagnosis (in years), sex, therapy (pentoxifyllin application, low-dose steroids, high-dose steroids), exploratory tympanotomy with sealing of the round and oval window niche (yes/no), tinnitus (yes/no), vertigo (yes/no), spontaneous nystagmus (yes/no), caloric test result (pathological/normal), positional nystagmus (yes/no), nicotine abuse (yes/no), objectively identifiable perilymph fistula (yes/no), previous hearing loss (yes/no), and fibrinogen level (elevated: fibrinogen level > 300 mg/dl, normal: fibrinogen level ≤ 300 mg/dl).

### Statistical analyses

Descriptive statistical analyses were carried out computing medians and interquartile ranges (IQR), absolute and relative frequencies, and Spearman’s correlation coefficient [50]. Fisher’s exact test and the chi^2^-test were used to assess the relationship between two categorical variables (e.g. between sex and hearing recovery), where Fisher’s exact test was used for two binary variables [51]. The relationships between categorical and quantitative variables (e.g. between sex and magnitude of hearing recovery) were evaluated by the Kruskal-Wallis-test [52]. Multivariate linear and logistic regression models were built to reassess the results of the univariate analyses. Model building was carried out by means of backward variable selection based on Akaike’s Information Criterion [53]. Coefficients from linear regression were checked by the Wald-test. Odds Ratios and referring confidence intervals were derived from the logistic regression coefficients. All statistical computations were done using R 3.1.2 [54]. Please note that all inferential statistics, in particular *p*-values, were intended to be exploratory (hypotheses-generating), not confirmatory. Therefore, *p*-values are to be interpreted in Fisher’s sense, representing the metric weight of evidence. Since a global level of significance was not controlled, *p*-values smaller than 5% are termed noticeable instead of significant.

## Results

### Patients’ basic clinical characteristics

By critical chart review we were able to identify 198 patients with hearing loss in accordance to our inclusion criteria. The time delay between onset of symptoms and start of the therapy was one to seven days. Some of the examined factors could not be evaluated in all 198 patients (Table 1). Of the 198 patients 106 were males (53.5%) and 92 were females (46.5%). The age ranged from 7 to 93 years with a mean age of 53.9 years (standard deviation: 18.0 years). Onehundredfive patients had hearing loss of the right side (53.0%) and 93 patients (47.0%) of the left ear. Accompanying tinnitus or vertigo was reported by 76.9% and 31.6% of patients. Hence, spontaneous nystagmus could only be detected in 12.8% of cases.

**Table 1:** Influence of clinical parameters on the chance of hearing recovery

Parameter		Recovery	No recovery	*p*-value
Median age (IQR)		56 (25)	58 (24)	0.178
Sex	Female	23 (25%)	69 (75%)	0.007
	Male	46 (43%)	60 (57%)	
Type of therapy	pentoxifyllin	6 (30%)	14 (70%)	0.104
	low-dose steroids	17 (26%)	49 (74%)	
	high-dose steroids	46 (41%)	66 (59%)	
Tympanotomy	No	12 (24%)	39 (76%)	0.061
	Yes	57 (39%)	90 (61%)	
Tinnitus	No	12 (29%)	30 (71%)	0.460
	Yes	51 (36%)	89 (64%)	
Vertigo	No	51 (40%)	77 (60%)	0.103
	Yes	16 (27%)	43 (73%)	
Spontaneous nystagmus	No	61 (37%)	103 (63%)	0.169
	Yes	5 (21%)	19 (79%)	
Caloric test result	Normal	52 (38%)	86 (62%)	0.051
	Pathological	5 (17%)	24 (83%)	
Positional nystagmus	No	16 (32%)	34 (68%)	0.793
	Yes	9 (38%)	15 (62%)	
Nicotine abuse	No	33 (31%)	75 (69%)	0.171
	Yes	34 (40%)	50 (60%)	
Perilymph fistula	No	45 (39%)	71 (61%)	0.737
	Yes	4 (44%)	5 (56%)	
Previous hearing loss	No	58 (35%)	109 (65%)	1.000
	Yes	9 (36%)	16 (64%)	
Elevated fibrinogen level	No	32 (32%)	68 (68%)	0.809
	Yes	8 (35%)	15 (65%)	

Vestibular testing showed a positional nystagmus in 32.4% of cases and pathological results in caloric testing in 17.4% of cases, while 5.4% of all tested patients had complete canal paresis and 12.0% had a directional preponderance. Thirteen percent of all patients reported similar events on the affected side in the past and 9.1% of patients stated that they had hearing loss or tinnitus on the contralateral side in the past. Daily alcohol abuse or nicotine abuse was documented in 3.4% or 43.8% of patients, respectively. Elevated fibrinogen plasma levels could be detected in 18.7% of cases. Patients with abnormalities in the MRI, as a sign for a retrocochlear origin of the hearing loss, or serological tests were excluded from further analysis, as well as patients with a possible indication of a non-idiopathic origin of hearing loss.

### Applied drug therapy

Special interest was given to the method of the applied drug therapy. The vast majority of SSHL patients received systemic steroids over eight days. Primarily dependent on patients’ health status three different schemes were applied, two with a combination of steroids and pentoxifyllin, and one containing only pentoxifyllin. The dosage of pentoxifyllin was identical in all three therapy regimens (Table 2). A sole administration of Pentoxifyllin, which was applied in cases of uncontrolled diabetes or other underlying disease with contraindication for prednisolone, was done in 10.1%. A low-dose steroid scheme was applied in 33.3% and a high-dose steroid scheme in 56.6% of cases (Table 2).

**Table 2:** Steroid and pentoxifyllin application schemes

Day of therapy	Prednisolone (mg)		Pentoxifyllin (mg)
	Low-dose	High-dose	
1	200	1000	100
2	200	800	100
3	150	600	150
4	150	500	300
5	100	300	300
6	100	200	300
7	75	100	300
8	50	75	300

### Surgical intervention

In case of existing criteria which provide an indication of a possible (PLF) patients were offered an explorative tympanotomy. During this procedure the round and oval window were screened for the presence of a PLF and were covered with soft tissue regardless of a visible defect. An exploratory tympanotomy was conducted in 147 of the 198 included patients (74.2%), whereby only in 7.2% of these patients a PLF could reliably be detected by the surgeon.

### Predictors of the chance of a hearing recovery (all patients)

Table 1 lists the results regarding the influence of clinical parameters on the chance of a hearing recovery. The only noticeable clinical parameter found was the gender, with men having a 2.3 times higher chance of a hearing recovery (Odds Ratio = OR = (46/60)/(23/69) = 2.3). Further clinical parameters that offered a small *p*-value were the tympanotomy (OR(yes vs. no) = 2.06) and the caloric test result (OR(normal vs. pathological) = 2.90). Furthermore, the *p*-values suggest that the type of therapy and the presence/absence of vertigo might possibly be predictors of a hearing recovery.

Starting from a multivariate logistic regression model that includes all these variables as covariates, the AIC-based backward variable selection delivered a model that includes the three covariates caloric test result, sex, and type of therapy. Here the caloric test result was the only noticeable predictor (Wald-test: *p* = 0.022), with an estimated OR (normal vs. pathological caloric test result) of approximately 3.4. Yet the model also indicates that the chance of a hearing recovery is possibly higher for male patients and for patients who received a high-dose steroid therapy, compared to patients with a pentoxifyllin application (data not shown).

### Predictors of the magnitude of a hearing recovery (all patients)

**Fig. 1:**
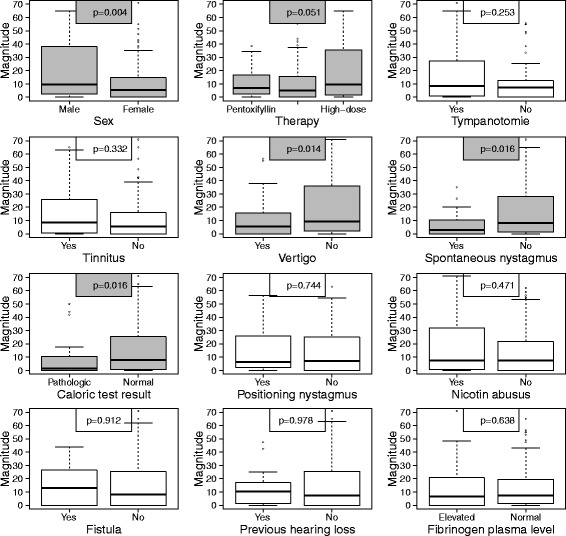
Influence of clinical parameters on the magnitude of a hearing recovery in all patients (*N* = 198, univariate analyses). The upper and the lower boundary of the boxes indicate the 75^th^ and the 25^th^ percentile, respectively. The line within the boxes marks the median. The whiskers below and above the box indicate the 10^th^ and the 90^th^ percentile. In statistical relevant issues the boxes are coloured grey, otherwise blank

The influence of the considered clinical parameters on the magnitude of a hearing recovery was investigated by means of boxplots, see Fig. 1. A noticeably higher magnitude of hearing recovery could be observed for men and for patients without vertigo, without spontaneous nystagmus, and with a normal caloric test result (compared to the respective counterparts). Furthermore, the small *p*-value suggests that a high-dose steroid therapy possibly leads to a higher magnitude than a low-dose steroid or pentoxifyllin therapy. A noticeable correlation between the patient’s age and the magnitude could not be observed (Spearman correlation:−0.08, *p* = 0.235).

**Fig. 2:**
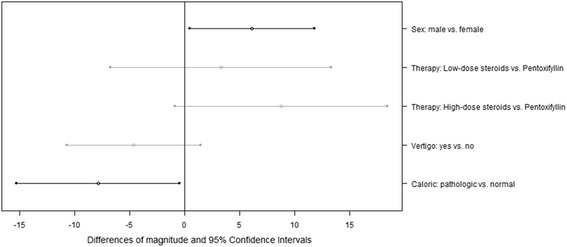
Differences of the magnitude of hearing recovery and related 95% Confidence Intervals for several clinical parameters, derived by multivariate linear regression, in all patients (*N* = 198)

In order to investigate the noticeable variables simultaneously as to differences of the magnitude, they are taken as possible covariates of a multivariate linear regression model. Since almost all patients who received a tympanotomy also received a cortisone therapy (i.e. low-dose or high-dose steroids), the variable tympanotomy was also considered as possible covariate of the model. The AIC-based backward selection delivered a model that includes the four covariates sex, therapy, vertigo, and caloric test result. The estimated model coefficients equate to the differences in magnitude between the categories, e.g. between men and women. The differences and the referring 95% CIs are depicted in Fig. 2.

Here the variables sex and caloric test result were noticeable predictors (Wald-test: *p* = 0.036 and *p* = 0.039). The magnitude was estimated to be around 6 dB higher for men and around 8 dB lower for patients with pathologic caloric test result, in comparison to the respective counterpart. The model also suggests that the magnitude is possibly lower for patients with vertigo and that the high-dose therapy possibly leads to a better outcome than the pentoxifyllin application: the magnitude was estimated to be around 9 dB higher for patients with high-dose steroid therapy (Wald-test: *p* = 0.076).

### Predictors of the magnitude of hearing recovery in patients with recovery

**Fig. 3:**
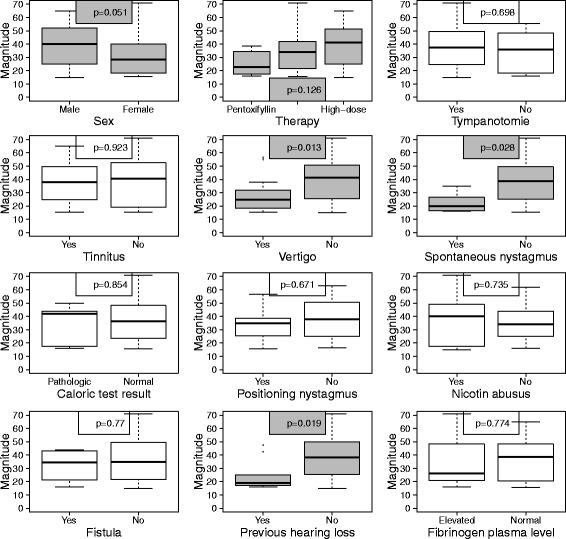
Influence of clinical parameters on the magnitude of a hearing recovery for the subgroup of patients who had a hearing recovery (*N* = 69, univariate analyses). The upper and the lower boundary of the boxes indicate the 75th and the 25th percentile, respectively. The line within the boxes marks the median. The whiskers below and above the box indicate the 10th and the 90th percentile. In statistical relevant issues the boxes are coloured grey, otherwise blank

We also investigated the subgroup of patients who actually had a hearing recovery (*n* = 69) in order to find possible predictors for the magnitude of a hearing recovery (Fig. 3).

**Fig. 4:**
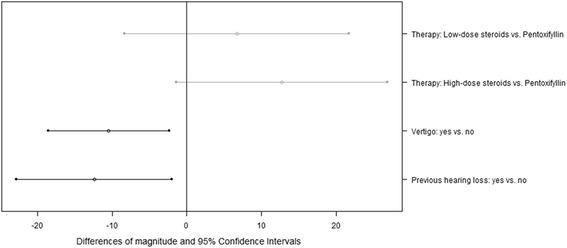
Subgroup of patients who attained a hearing recovery (*N* = 69): differences of the magnitude of hearing recovery and associated 95% Confidence Intervals for several clinical parameters, derived by multivariate linear regression

For this subgroup, an influence of the caloric test result on the magnitude of hearing recovery could not be observed. Instead, the magnitude was noticeably higher if no hearing loss had occurred before. The multivariate linear regression model that results from AIC-based backward selection includes the three covariates therapy, vertigo, and previous hearing loss. The model coefficients and the 95% CIs are shown in Fig. 4. Vertigo and previous hearing loss were found to be associated with a noticeably lower magnitude (Wald-test, *p* = 0.014 and *p* = 0.023, estimated differences: 10 dB and 12 dB). The model also indicates that patients who received a high-dose steroid therapy possibly attain a higher magnitude of hearing recovery than patients with a pentoxifyllin application; the magnitude was estimated to be approximately 13 dB higher for patients with a high-dose steroid therapy (Wald-test: *p* = 0.083).

## Discussion

The etiology and mechanisms that cause SSHL are still unknown. Even the natural course of SSHL in patients has been still a matter of discussion in the last decade since the rate of spontaneous recovery has been estimated between 30% and 60% [24–26]. Moreover, the majority of patients do not completely recover after SSHL, although treated by drugs and the knowledge about prognostic factors is very limited. The actual treatment guidelines for SSHL include the use of systemic glucocorticoids, Fibrinogen/LDL apheresis and administration of steroids via intratympanic routes [19–23]. Yet, the therapeutic benefit of some of these treatment options remains unclear since results from randomised controlled trials are still missing. Besides classical drug therapy with steroids most task forces recommend exclusion of a PLF by an exploratory tympanotomy especially in pancochlear SSHL with additional symptoms like disequilibrium or nystagmus [27, 28]. This procedure is an uncomplicated and fast surgical intervention, but, detection of a PLF is technically difficult and dependent on the investigator. In addition there is no clear evidence of therapeutic efficacy in patients without classical clinical picture and history of PLF [27].

The aim of this study was to investigate possible positive or negative predictors of hearing recovery in patients with severe SSHL. We could demonstrate a clear benefit in hearing recovery for patients missing subjective and objective symptoms of vestibular dysfunction accompanying hearing loss. Regarding all patients, the chance for hearing recovery as well as the magnitude of hearing recovery demonstrated a dependency on such vestibular parameters. While the absence of vertigo or spontaneous nystagmus were only associated with higher magnitude of recovery in univariate analysis, normal caloric testing proofed to be the decisive key variable for a higher chance of hearing recovery as well as for a higher magnitude of recovery in both, univariate and multivariate analysis. However, looking just at the patients with actual recovery, absence of spontaneous nystagmus was associated with a higher magnitude of recovery in univariate analysis and absence of vertigo was associated with a higher magnitude of recovery in univariate and multivariate analysis.

In the last few decades many studies analysed possible predictors of hearing recovery in SSHL [29–45]. The association between the subjective perception of vertigo and worse outcome could be demonstrated by several studies [32, 33, 36–45]. But, the association between objective calorimetric tests and hearing outcome was only investigated by a few [29, 31, 32]. There are numerous differences between these studies and the one presented here; mostly important are the type of hearing loss and the time from onset to diagnosis. Nevertheless, all studies could clearly demonstrate a negative association between abnormalities in caloric testing and hearing recovery. The disproportion between the relevant number of patients with subjective complaint of vertigo and lack of pathological results in vestibular testing in our study might be explained by the fact that a lesion in the vestibular end organs was so small as to be detectable by routine tests or so transient that the lesion had normalized by the time of testing [45]. Due to the low number of cases with complete canal paresis and directional preponderance we gave up a subgroup analysis. The reason for the negative impact of pathological results in caloric testing on hearing recovery after SSHL might be due to a special etiology, especially a viral infection or viral reactivation. In terms of a labyrinthitis, viral agents are able to cause severe and irreversible hearing loss [38]. Yet, all patients included in this study had to have a negative serological test for HSV and VZV. Of course, this does not rule out all possible viral agents that may cause a labyrinthitis [38].

Unfortunately, because of insufficient documentation we were not able to do a subgroup analysis on hearing recovery in dependency on time delay from onset of symptoms like it was done by the other study groups. Age was no relevant predictor in the here presented study, as well as in several other studies, but, also in contrast to other studies [29, 30, 32–37]. Surprisingly, our own data also indicate that males have a better prognosis than women. This could be shown for the chance of hearing recovery in univariate analysis and for the magnitude of recovery in univariate and multivariate analysis. Within the group of patients with actual recovery we only found a borderline significant relation between male gender and improved outcome in univariate analysis. An association between gender and outcome after SSHL was detected by only two other study groups so far [30, 46]. The fact that females had worse hearing recovery could not be attributed to influences of other parameter since the relationship stayed significant in multivariate analysis and possible negative predictors like age, fibrinogen plasma levels and rate of cardiovascular disease showed no differences to male patients (data not shown).

Generally, the classification of improvement shows a great variation in the above mentioned studies. Many study groups use their own developed classification system, thus reducing comparability between the different studies, if not making it impossible. We used the Siegel’s criteria for classifying hearing recovery, an internationally accepted classification system, which is, nevertheless, not often used [35, 40, 49].

Interestingly, considering all patients studied, a previously occurred hearing loss had no relevant prognostic importance. While in patients with documented improvement the magnitude of hearing recovery was significantly higher if such an event was not reported by the patients, in univariate as well as in multivariate analysis. To be noted, even though 13% of our patients had recurrent hearing loss none of these patients had a history or symptoms for diagnosing Ménière’s disease and a vestibular schwannoma could be ruled out by MRI. Similar findings demonstrating poorer prognosis after recurrent episode of SSHL could also be shown by others [47, 48]. The reasons for this association could not be clarified yet.

Hearing loss therapy is an intensively debated field. The vast majority of the patients in this study were treated with intravenous steroids. Despite intensive review of patients’ medical chart we could not identify decision criterion for applying low-dose or high-dose steroids. There was a clear trend towards improved outcome in patients receiving high-dose steroids, but, no significant relationship. The question if steroids or the dose of the administered steroids have important influence on hearing recovery remains controversial [21–23, 29, 44, 55–57].

Although, nineteen percent of our patients had elevated fibrinogen plasma levels at first presentation none of them received apharesis. The therapeutic value of fibrinogen and LDL apharesis in SSHL patients seems to be comparable to that of standard regimen with glucocorticoids [20, 21]. Latest treatment guidelines do not even mention apheresis as first line treatment option [56].

A novel treatment option is application of steroids through intratympanic routes [19]. Reliable data on the effectiveness of this therapy have only recently been published. Hence, this was no therapeutic option for our patients.

Many of the patients included in this study underwent an explorative tympanotomy in order to rule out a PLF. The reason for the surprisingly high number of operative interventions could not be further elucidated despite the intensive research of the patients’ medical charts. A PLF could only be detected in 7%. Comparing both collectives, patients receiving tympanotomy and those who did not, there was no significant difference in hearing recovery between these two groups. To our surprise, even if a PLF could be seen by the surgeon, the coverage of it did not influence hearing recovery. This is in accordance to findings of others [58].

Since we only evaluated test results prior to therapy and the vast majority of our patients initially showed up outside regular working hours we unfortunately were not able to use data from other audiometric tests, e.g. speech audiometry to evaluate hearing recovery [20].

## Conclusions

Altogether we were able to demonstrate that the presence of symptoms or objective criteria of vestibular dysfunction in patients with SSHL are associated with worse hearing recovery. We therefore think that patients with hearing loss and additional vestibular dysfunction represent a subentity of SSHL patients. This might indicate a special disease entity with an own pathophysiological background, e.g. viral labyrinthitis. A similar context could exist for hearing loss associated with previous events. Unfortunately, due to the retrospective character of the presented study there is no sufficient data available to allocate the origin of these potential sub-entities to an individual background, namely vascular or inflammatory [59]. Furthermore, the retrospective analysis of numerous, potentially relevant parameters leads to a partially incomplete data set which presents a potential bias and limits the validity of the presented data. We thus plan a prospective randomized controlled trial in order to clarify possible causal links. Even though our data concerning dosage of steroids and outcome is inconsistent we would recommend applying a high-dose regimen, if one considers steroids in SSHL treatment.
